# Neural Crest Cells Contribute an Astrocyte-like Glial Population to the Spleen

**DOI:** 10.1038/srep45645

**Published:** 2017-03-28

**Authors:** Amanda J. Barlow-Anacker, Ming Fu, Christopher S. Erickson, Federica Bertocchini, Ankush Gosain

**Affiliations:** 1 Department of Surgery, University of Wisconsin School of Medicine and Public Health, Madison, Wisconsin, United States of America; 2 Division of Pediatric Surgery, Department of Surgery, University of Tennessee Health Sciences Center, Memphis, Tennessee, United States of America; 3 Instituto de Biomedicina y Biotechnologia de Cantabria, Santander, Spain; 4 Children’s Foundation Research Institute, Le Bonheur Children’s Hospital, Memphis, Tennessee, United States of America

## Abstract

Neural crest cells (NCC) are multi-potent cells of ectodermal origin that colonize diverse organs, including the gastrointestinal tract to form the enteric nervous system (ENS) and hematopoietic organs (bone marrow, thymus) where they participate in lymphocyte trafficking. Recent studies have implicated the spleen as an anatomic site for integration of inflammatory signals from the intestine with efferent neural inputs. We have previously observed alterations in splenic lymphocyte subsets in animals with defective migration of NCC that model Hirschsprung’s disease, leading us to hypothesize that there may be a direct cellular contribution of NCC to the spleen. Here, we demonstrate that NCC colonize the spleen during embryogenesis and persist into adulthood. Splenic NCC display markers indicating a glial lineage and are arranged anatomically adjacent to blood vessels, pericytes and nerves, suggesting an astrocyte-like phenotype. Finally, we identify similar neural-crest derived cells in both the avian and non-human primate spleen, showing evolutionary conservation of these cells.

The spleen functions to filter circulating blood, removing senescent erythrocytes as well as to present circulating antigens to cells of the immune system. Several recent studies have implicated the spleen as an anatomic site for integration of inflammatory signals from the intestine with efferent neural inputs, resulting in modulation of the host response to inflammation^1^. In experimental models of inflammatory bowel diseases (e.g. Crohn’s Disease and Ulcerative Colitis), the vagus nerve exhibits a tonic inhibitory effect on macrophage elaboration of pro-inflammatory cytokines through a cholinergic a7nAChR-dependent pathway^2^. Additionally, the splenic nerve has been implicated in this cholinergic anti-inflammatory pathway^3^. However, the cellular mechanism by which these signals are integrated is unknown.

Neural crest cells (NCC) are a population of cells, found only in vertebrates, that arise from the border of the neural plate and non-neural ectoderm during development^4^. Early, migrating NCC are multipotent, and are considered stem cell-like because of their capacity for self-renewal. NCC migrate along distinct pathways based on their level of origin along the body axis (e.g. cranial, vagal, trunk and sacral) and give rise to diverse cell lineages throughout the body^5^. Neural crest-derived cells include melanocytes, bony and cartilaginous structures of the craniofacial skeleton, cardiac outflow tract, sensory and sympathetic ganglia, Schwann cells, and the ENS. In the ENS, NCC cells colonize the gut and differentiate to form neurons and enteric glial cells (EGC). Within the gut, EGC resemble astrocytes of the central nervous system (CNS) in that they envelop enteric neuronal cell bodies and axons and their processes extend into the mucosa^6^,^7^. Recently, this relationship has been shown to be functionally significant, with enteric blood vessel endothelial cells surrounded by EGCs and pericytes serving as a gut-vascular barrier akin to the blood-brain barrier in the CNS^8^. In addition to their well-characterized role in the development of the ENS, recent studies have demonstrated NCC colonization of hematopoietic organs (bone marrow), as well as secondary lymphoid organs (thymus)^9^,^10^,^11^,^12^. In these organs, NCC-derived cells participate in lymphocyte trafficking through site-specific mechanisms^9^,^13^.

Hirschsprung’s disease (HSCR, Online Mendelian Inheritance in Man #142623) results from defective NCC migration to, and colonization of, the hindgut during embryonic development^14^. Most commonly, HSCR results from mutations in *Rearranged during transfection (Ret*) or *Endothelin Receptor B (EdnrB*), both of which are required for NCC migration^15^. The resulting absence of enteric neurons in varying lengths of the distal bowel results in a spectrum of symptoms postnatally, depending on the length of the bowel affected, and includes constipation, vomiting, abdominal distension and Hirschsprung’s-associated enterocolitis (HAEC), a potentially life-threatening form of intestinal inflammation that affects newborn infants with Hirschsprung’s disease^16^. Multiple recent studies have noted small splenic size in murine models of Hirschsprung’s disease^17^,^18^. Using the neural crest conditional deletion of *EdnrB (EdnrB*^*NCC*−/−^) model of HSCR^19^, we have previously observed impaired cellular immunity during HAEC, including decreased levels of secretory immunoglobulin A within the small bowel lumen and decreased numbers of the specific population of B lymphocytes responsible for immunoglobulin production (mature B lymphocytes) within the Peyer’s Patches (PP) of the small intestine 18. These mucosal immune alterations in the gut were accompanied by an increased fraction of mature B lymphocytes within the spleens of HSCR animals, suggesting impaired trafficking of these cells from the spleen to the PP.

Based on the known role of NCC-derived cells in regulating lymphocyte trafficking in thymus and bone marrow and our prior findings of altered lymphocyte populations within the *EdnrB*^*NCC*−/−^ spleen, we sought to test the hypothesis that there is a direct cellular contribution of the neural crest to the spleen. Here, by modification of our previously described model to enhance visualization of migrating NCC, we have made the novel observation that neural crest-derived cells are present in the spleen during embryogenesis and persist into adulthood. We demonstrate the time-course of entry of these neural crest-derived cells to the spleen, their expression of glial lineage markers, and association with blood vessels, pericytes and nerves, suggesting an astrocyte-like phenotype. We additionally note alterations in known lymphocyte trafficking cues in the *EdnrB*^*NCC*−/−^ spleen, as well as presence of neural-crest derived cells in the avian and non-human primate spleen, suggesting an evolutionary conservation of these cells.

## Results

### *EdnrB* ^*NCC*+/−^ and *EdnrB* ^*NCC*−/−^ animals model HSCR and can be used to visualize NCC

**Figure 1:**
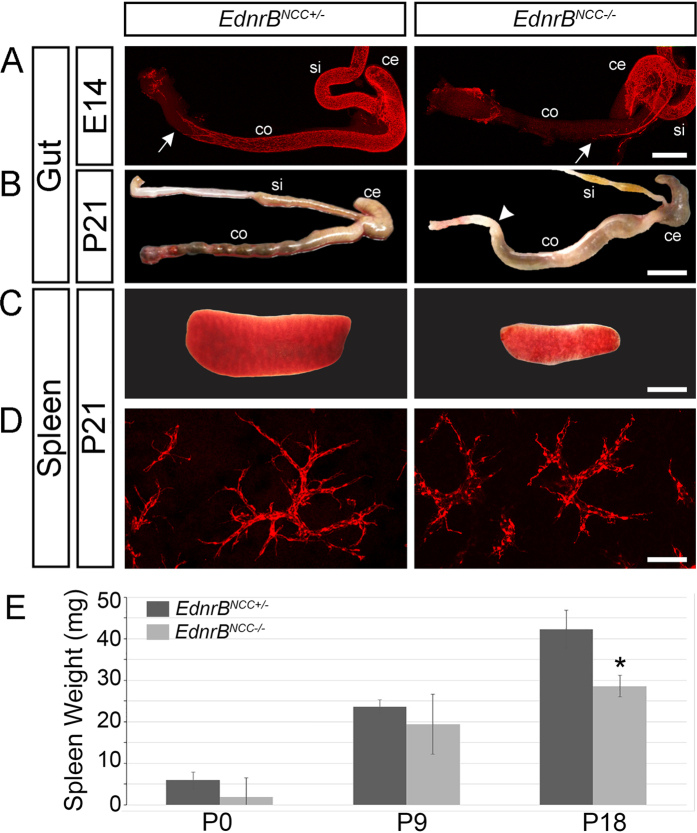
Embryonic and postnatal hindgut and spleens of *EdnrB*^*NCC*+/−^ and *EdnrB*^*NCC*−/−^ animals. (**A**) tdTomato visualization of NCC in the small intestine and colon shows delayed colonization of the colon in *EdnrB*^*NCC*−/−^ *animals* compared to *EdnrB*^*NCC*+/−^, with the migratory wavefront of NCC marked by white arrows at E14.5. (**B**) Aganglionosis in the distal colon of *EdnrB*^*NCC*−/−^ *animals* at P21 causes functional obstruction (marked by white arrowhead). Normal, pelleted stool is seen in the distal *EdnrB*^*NCC*+/−^ colon. (**C**) Reduced splenic size of *EdnrB*^*NCC*−/−^ compared to *EdnrB*^*NCC*+/−^ animals at P21. (**D**) tdTomato expressing NCC in *EdnrB*^*NCC*+/−^ and *EdnrB*^*NCC*−/−^ spleens at P21. (**E**) Spleens were harvested from *EdnrB*^*NCC*+/−^ and *EdnrB*^*NCC*−/−^ animals at P0, P9 and P18 and weighed. (*p < 0.05). Scale bars: A 400 μm, B 1 cm, C 4 mm and D 100 μm.

Mice carrying a NCC specific deletion of Endothelin Receptor B (*EdnrB*^*flex3/flex3*^) were mated with *TgWnt1-Cre*/+; *Rosa26*^*floxStop/tdTomato*^ animals to produce mice with either a heterozygous (*EdnrB*^*flex3*/+^) or homozygous deletion of *EdnrB (EdnrB*^*flex3/flex3*^). Throughout the manuscript these mice will be described as *EdnrB*^*NCC*+/−^ and *EdnrB*^*NCC*−/−^, respectively. *EdnrB*^*NCC*−/−^ have previously been shown to result in a HSCR phenotype caused by failure of NCC migration to the distal hindgut^19^. These animals additionally develop HAEC post-natally, with death from enterocolitis occurring in the fourth week of life^18^,^20^. By targeting the tdTomato fluorophore, which is stable following fixation of tissues, to the ROSA26 locus, NCC are readily visualized^21^. Using this model, we harvested distal small bowel and colon from embryonic (E) day 14 *EdnrB*^*NCC*+/−^ and *EdnrB*^*NCC*−/−^ animals and visualized NCC colonization by whole mount immunofluorescence. We observed the failure of NCC migration into the distal hindgut in *EdnrB*^*NCC*−/−^ animals (Fig. 1A), consistent with our prior reports with other fluorophores in this model^22^. Examination of post-natal (P) day 21 bowel revealed normal, pelleted stools in the distal colon of *EdnrB*^*NCC*+/−^ animals, while the *EdnrB*^*NCC*−/−^ animals demonstrated distal bowel obstruction, with proximal colonic dilation and a large stool burden in the colon, consistent with a HSCR phenotype (Fig. 1B).

### NCC are present within the spleen of *EdnrB* ^*NCC*+/−^ and *EdnrB* ^*NCC*−/−^ animals in similar numbers

Two previous studies have demonstrated similar immune defects in the spleens of both conventional and NCC-conditional *EdnrB* mutant animals^18^,^23^. Additionally, small splenic size has been noted in the conventional *EdnrB* deletion model^17^,^24^. Based on this, we hypothesized that there may be a direct contribution of NCC to the spleen. We examined the spleens of *EdnrB*^*NCC*+/−^ and *EdnrB*^*NCC*−/−^ animals and confirmed the finding of small splenic size in our conditional deletion model (Fig. 1C). We then examined spleens from animals at P0, P9, and P18 (Fig. 1E). We noted no differences in splenic weight between genotypes at the earlier time points, however, by P18 the spleens of *EdnrB*^*NCC*−/−^ mice were significantly smaller than their heterozygous littermates (42.2 ± 2.9 mg vs. 28.6 ± 1.7 mg, *p* = 0.016).

Close examination of the spleen revealed the novel finding of robust presence of tdTomato NCC throughout the organ in both *EdnrB*^*NCC*+/−^ and *EdnrB*^*NCC*−/−^ animals (Fig. 1D). The aganglionic colon HSCR phenotype is due to a failure of NCC migration to the distal hindgut. This has been shown to result, at least in part, from a non-receptive environment in the hindgut^25^. It has additionally been hypothesized that there is a decreased number of NCC available to colonize the gut in HSCR^26^. To determine if the previously described alterations in splenic size and lymphocyte populations in the *EdnrB*^*NCC*−/−^ animal^18^ may be related to a decreased number of NCC colonizing the spleen, we examined P0 and P21 *EdnrB*^*NCC*+/−^ and *EdnrB*^*NCC*−/−^ spleens. We used immunofluorescence to determine the density of tdTomato NCC in splenic sections. At P0 there were 15.78 ± 0.95 cells/mm^2^ in the *EdnrB*^*NCC*+/−^, and 16.54 ± 0.67 cells/mm^2^ in the *EdnrB*^*NCC*−/−^ (*p* = 0.55). At P21 there were 13.37 ± 1.33 cells/mm^2^ in the *EdnrB*^*NCC*+/−^ and 18.72 ± 2.6 cells/mm^2^ in the *EdnrB*^*NCC*−/−^ (*p* = 0.15). Combined with data on splenic size, this suggests that there are no significant differences in the number of NCC between the *EdnrB*^*NCC*+/−^ and *EdnrB*^*NCC*−/−^ spleen through the early post-natal period.

### NCC expressing *EdnrB* enter the spleen during embryonic development

**Figure 2:**
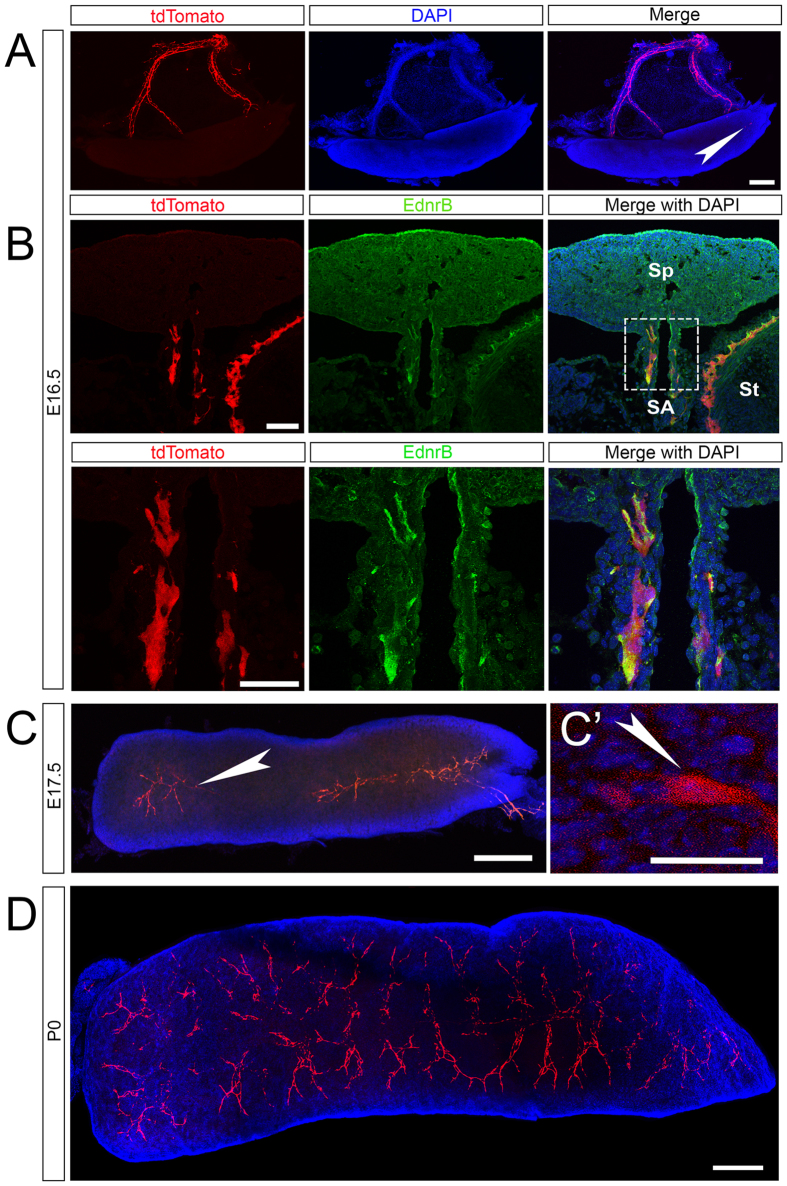
Identification of entry of tdTomato expressing NCC into the spleen. (**A**) Whole mount preparation of the spleen and hilar structures at E16.5 shows numerous NCC on the hilum and a small number within the spleen. (**B**) Upper panel: 16 μm section showing tdTomato NCC entering the spleen (Sp) along the hilum (splenic artery SA, stomach St) express EdnrB (yellow in merged panels). Lower Panel: High magnification of boxed region in upper panel. (**C**) At E17.5, tdTomato NCC enter the spleen from the hilum and colonize the organ. (C’) High magnification view of a single splenic NCC at E17.5. (**D**) By P0, large numbers of NCC have colonized the spleen in an arborized pattern. Arrowheads (white) highlight individual splenic NCC. Scale bars: A 200 μm, B 50 μm, C 200 μm, C’ 20 μm, D 200 μm.

To better understand the role of NCC in the spleen, we sought to determine the time course of their entry. We examined whole mount spleens from *EdnrB*^*NCC*+/−^ embryos. We noted NCC on the splenic artery at embryonic day (E) 14, with large numbers present by E16.5. We first noted entry of NCC into the spleen at E16.5 (Fig. 2A). Immunostaining for EdnrB along the splenic hilum demonstrated that NCC bound for the spleen express EdnrB (Fig. 2B), similar to those that colonize the bowel. Examination of the spleen at E17.5 demonstrated increased numbers of NCC entering the spleen in an arborized pattern, suggesting migration along the neurovasculature (Fig. 2C). By P0, there was robust colonization of the entire spleen by tdTomato NCC (Fig. 2D).

### NCC in the spleen are associated with the neurovasculature and display a non-Schwann cell glial lineage

**Figure 3:**
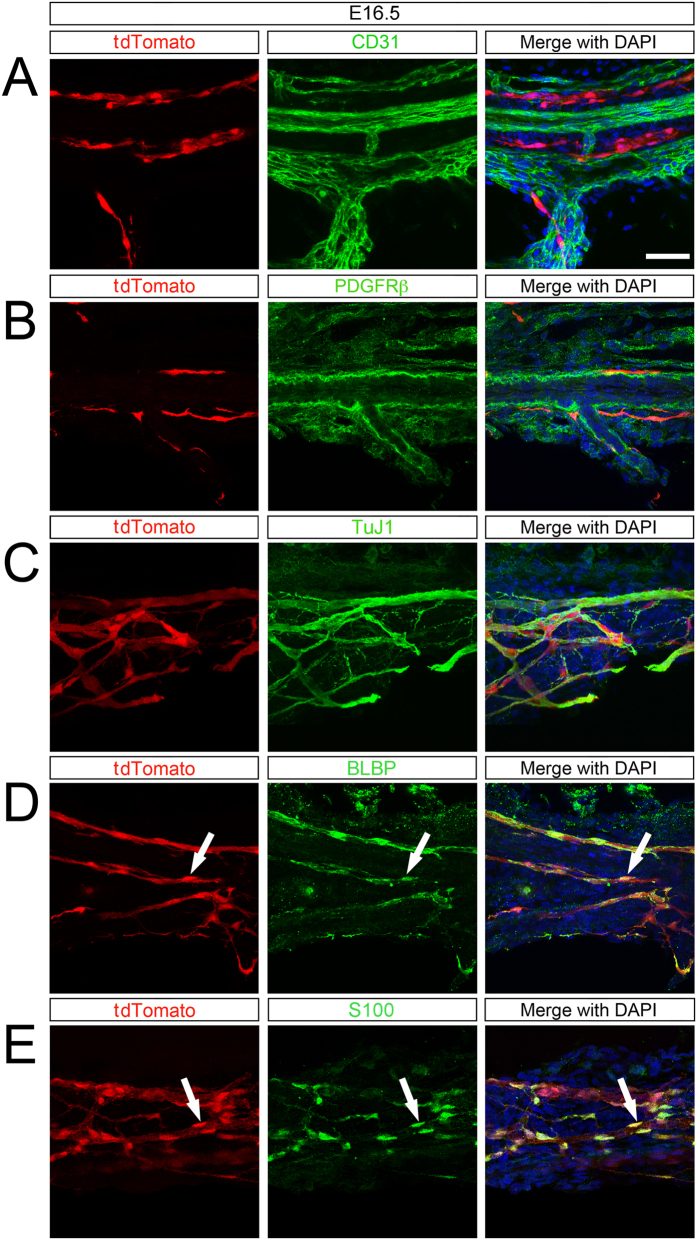
NCC along the splenic hilum at E16.5 express glial markers. (**A**) tdTomato NCC are located near, but are distinct from CD31 (endothelial cells) and (**B**) PDGFRβ (pericytes) which surround blood vessels. (**C**) tdTomato NCC are associated with TuJ1 (nerve fibers). (**D**) At E16.5, most tdTomato NCC express BLBP (early glial lineage) and (**E**) S100-β (glial lineage). Arrowheads (white) highlight double-labeled cells. Scale bar: 50 μm.

NCC have been shown to colonize other lymphoid organs, including the bone marrow and thymus^12^. In the bone marrow, NCC form mesenchymal stem cells that function to preserve the hematopoeitic stem cell niche^9^. NCC colonize the thymus where they form pericytes and participate in T lymphocyte trafficking^27^. To determine the cellular phenotype of NCC in the spleen, we performed immunohistochemistry at E16.5, P0, and P21 on spleens from *EdnrB*^*NCC*+/−^ animals. Staining of endothelial cells using CD31 revealed that NCC are located parallel, but not immediately apposed, to the splenic vessels at E16.5 (Fig. 3A). NCC are located adjacent to pericytes (PDGFR-β) which surround the blood vessels (Fig. 3B). Furthermore, NCC appear to be tightly associated with nerve fibers (TuJ1, Fig. 3C). At this stage, prior to entering the spleen, some NCC display the earliest glial marker BLBP (Fig. 3D) as well as the more mature glial marker S100-β (Fig. 3E).

At P0, when NCC have robustly colonized the spleen, we see a similar pattern of organization of NCC with respect to the vasculature and nerve fibers (Supplementary Figure 1A–D). By this stage, all splenic NCC express glial lineage markers (Supplementary Figure 1E,F) and continue to be associated with nerve fibers (Supplementary Figure 1D).

**Figure 4:**
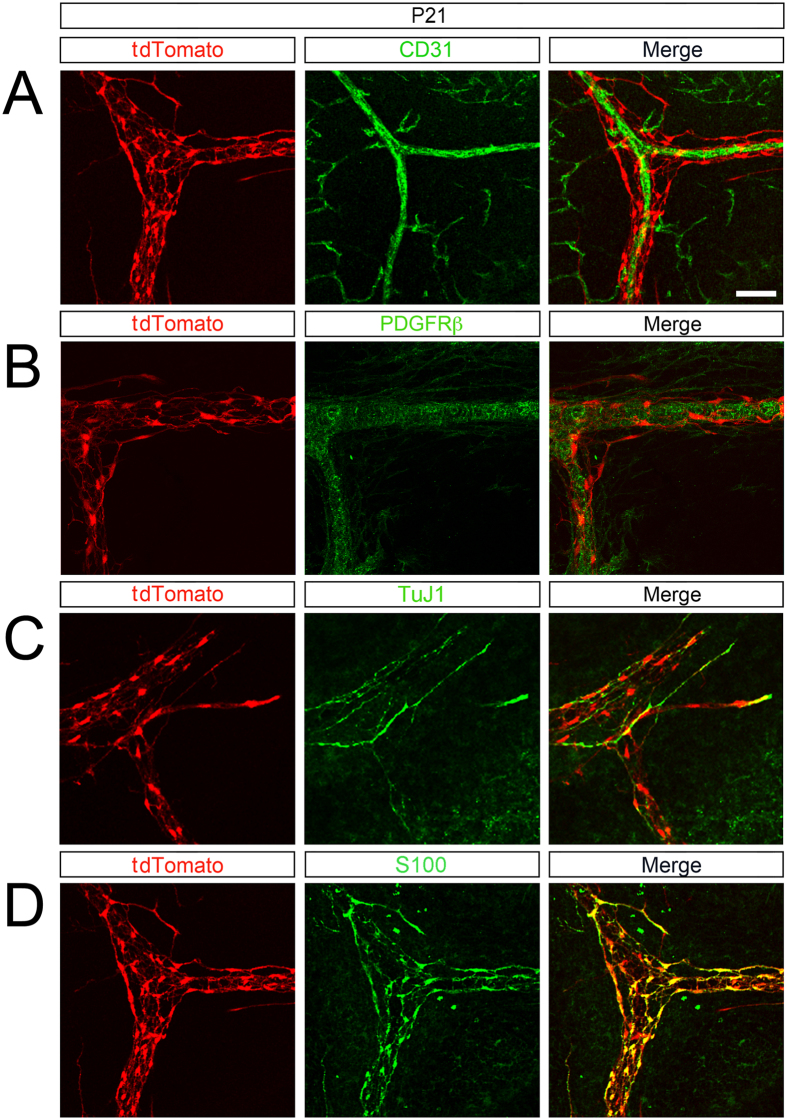
Splenic NCC within the spleen at P21 express glial markers. (**A**) Within the spleen, tdTomato NCC are present along blood vessels (CD31). (**B**) tdTomato NCC are associated with pericytes (PDGFRβ) and (**C**) nerve fibers (TuJ1). (**D**) At P21 all tdTomato NCC express S100-β (glial lineage). Scale bar: 50 μm.

The NCC contribution to the spleen is maintained postnatally (P21, Fig. 4). At this stage, splenic NCC cells continue to encircle the vasculature (Fig. 4A,B), are associated with nerve fibers (Fig. 4C) and display glial markers (Fig. 4D). Because of the proximity of NCC to TuJ1 + nerve fibers, we confirmed that splenic NCC are not neurons by staining for HuC/D (Supplemental Figure 5)^28^.

Schwann cells also derive from the neural crest^29^. Myelinating Schwann cells are glial lineage cells that surround peripheral nervous system axons and can be identified by staining for myelin-associated glycoprotein (MAG). We did not identify any MAG-positive staining of splenic NCC (Supplementary Figure 3). Schwann cell precursors (SCP) are a subpopulation of glial cells that are specified from the neural crest lineage^29^,^30^. Sox10, required for glial specification, is expressed in SCP with persistence of expression in mature myelinating and non-myelinating Schwann cells of the peripheral nervous system (PNS)^31^. We did not identify any Sox10 positive splenic NCC cells by immunohistochemistry (Supplementary Figure 4).

Together, these data suggest that, in contrast to NCC found in other hematopoeitic organs, splenic NCC form a glial lineage.

### Splenic NCC are present in avians and non-human primates

**Figure 5:**
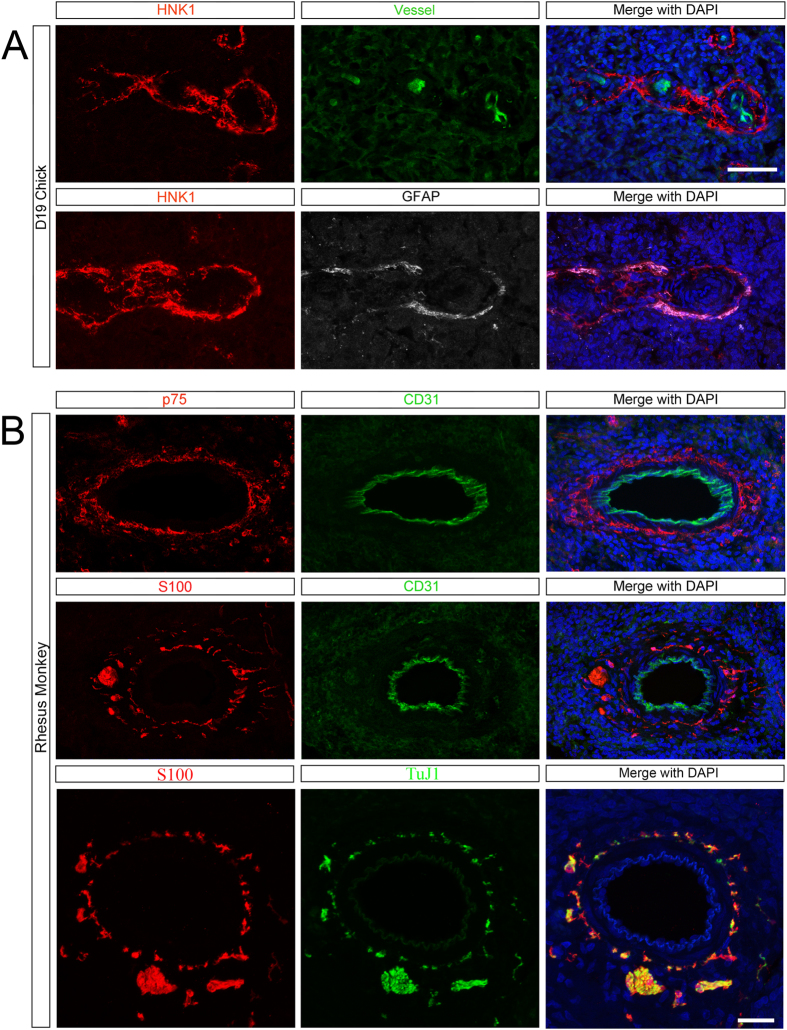
NCC are found in the spleens of avians and non-human primates. (**A**) NCC (HNK1 red) are located around blood vessels (endogenous GFP, green) and co-express GFAP (glial lineage, white) in the Day 19 embryonic chick spleen. (**B**) Top: NCC (p75, red) surround blood vessels (CD31, green) in the juvenile non-human primate spleen. Middle: Glial cells (S100-β, red) are located in a similar position to NCC. Bottom: Glial cells (S100-β, red) are found in proximity to TuJ1+ nerve fibers (green). See also Supplementary movie. Scale bar: 50 μm.

To determine if the contribution of NCC to the spleen is phylogenetically conserved, we examined the spleen of the developing chick and juvenile Rhesus macaque. By using a transgenic chick with ubiquitous expression of green fluorescent protein (GFP) we are able to visualize blood vessels without the need for intravascular injection of dye^32^. We found that the embryonic chick spleen contains HNK1-expressing NCC^33^ in a similar spatial organization relative to the blood vessels as we had observed in the mouse (Fig. 5A). Unlike the mouse, in which glial fibrillary acidic protein (GFAP) expression in glial cells is proportional to the degree of inflammation^34^, in the chick, GFAP can be used to distinguish glial cells from neuronal cells early in development^35^. We found that avian splenic NCC have coincident expression of GFAP (Fig. 5A), confirming their glial lineage.

In the juvenile Rhesus macaque, we observed p75-expressing neural crest-derived cells surrounding CD31 + blood vessels (Fig. 5B). Additionally, S100-β-expressing, glial linage cells are located in a similar position around these blood vessels and near TuJ1+ nerve fibers (Fig. 5B). Careful examination by 3D reconstruction demonstrates that NCC are in proximity to, but distinct from, TuJ1+ nerve fibers (Supplemental Fig. 2, Supplemental Movie).

Together, these data suggest that the NCC contribution to the spleen is evolutionarily conserved in vertebrates.

### Expression of S1P1 is reduced in *EdnrB* ^*NCC*−/−^ spleen

**Figure 6:**
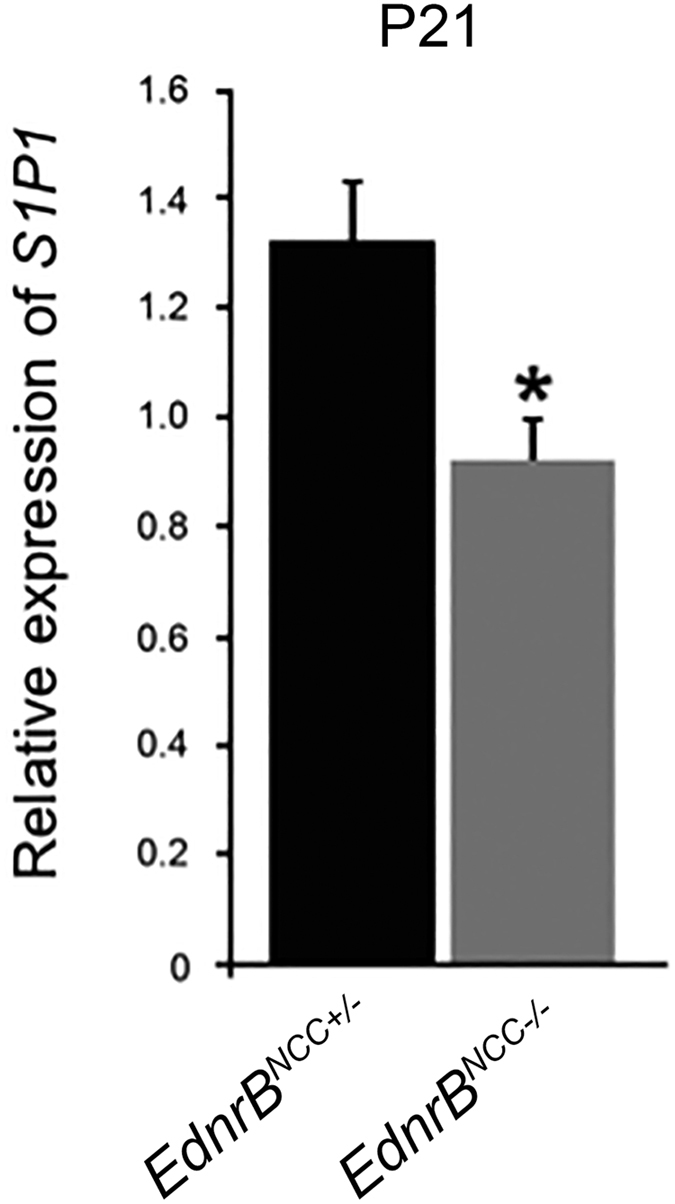
Expression of S1P1 is reduced in *EdnrB*^*NCC*−/−^ spleen. Relative expression of S1P1 is reduced in *EdnrB*^*NCC*−/−^ versus *EdnrB*^*NCC*+/−^ spleens at P21.

Marginal zone (MZ) B-lymphocytes encounter blood-borne pathogens and initiate an immune response^36^. We have previously noted decreased MZ B-lymphocytes in *EdnrB*^*NCC*−/−^ spleen just prior to HAEC^18^. B-lymphocyte localization to the MZ requires expression of the Sphingosine-1-Phosphate Receptor 1 (S1P1)^37^. In the thymus, Sphingosine-1-Phosphate (S1P) ligand is produced by NCC-derived cells^27^ and enteric glia have demonstrated responsiveness to S1P^38^. We measured relative expression of S1P1 in *EdnrB*^*NCC*+/−^ and *EdnrB*^*NCC*−/−^ spleen at P21, a time-point that is prior to the onset of HAEC in this model^20^, and noted decreased expression in *EdnrB*^*NCC*−/−^ animals (Fig. 6), suggesting a possible molecular mechanism for further functional investigation in this model.

## Discussion

We have demonstrated a novel contribution of NCC to the developing spleen. NCC migrate along the splenic artery and enter the hilum of the murine spleen by E16.5. Some of these NCC display glial-lineage markers (BLBP, S100-β) prior to entering the spleen. By P21, all splenic NCC express these markers and are closely associated with the vasculature, near endothelial cells and pericytes, as well as nerve fibers, in a spatial configuration that suggests an astrocyte-like identity. We have observed a similar contribution of NCC to the avian and non-human primate spleen, suggesting the importance of NCC in splenic development and/or function. Finally, we noted decreased expression of the lymphocyte trafficking receptor, S1P1, in *EdnrB*^*NCC*−/−^ spleen, suggesting a potential functional role for these NCC-derived cells in regulation of splenic lymphocyte trafficking.

In our previous studies of the NCC-conditional *EdnrB*^*NCC*−/−^ mouse model of HSCR/HAEC, we observed alterations in splenic lymphocyte populations and a small splenic size, indicating that lymphocyte trafficking is altered in this model^18^. Additionally, in the conventional *EdnrB*^*NCC*−/−^ model of HSCR/HAEC, transplant of *EdnrB*^*NCC*−/−^ bone marrow into WT recipients resulted in small splenic size^23^. Advances in transgenic mouse technology, knowledge of neural crest developmental markers, and the stability of fluorophores have allowed for precise lineage tracing of migrating NCC into diverse organs^12^,^26^,^39^,^40^. Of specific importance to the current study, use of these techniques has demonstrated that hematopoietic organs, including the bone marrow and thymus, contain NCC. NCC enter the thymus during embryonic development before E13.5, and form pericytes, which are closely associated with endothelial cells^11^,^39^. Thymic NCC-derived pericytes produce S1P, which modulates thymocyte egress into the blood^13^. In bone marrow, NCC contribute to the hematopoietic stem cell niche^9^,^41^,^42^. Similar to the splenic NCC, bone marrow NCC are associated with the vasculature. Bone marrow NCC-derived cells produce the cytokines Cxcl12 and stem cell factor that attract hematopoietic progenitor and stem cells^9^.

The spatial configuration of splenic NCC in relation to endothelial cells, pericytes and nerve fibers suggests an astrocyte-like identity, similar to that seen in the neurovascular unit (NVU) of the CNS. The blood-brain barrier, composed of endothelial cells with tight junctions, has been considered the primary determinant of molecular trafficking from the bloodstream in and out of the CNS^43^. The NVU was originally proposed as a framework to explain the coupling of neuronal activity (energy demand) to local blood flow (energy supply) in the brain^44^. This framework has evolved to include multiple cellular components that contribute to NVU structure and function, including endothelial cells, pericytes, astrocytes, microglia, and neurons. Importantly, this expanded view of the NVU accounts for the role that it and the blood brain barrier (as a component of the NVU) play in control of cerebrovascular blood flow, immune surveillance and the pathogenesis of a wide range of neurologic disease^43^,^45^. In the NVU, endothelial cells form a monolayer bound by tight junctions and resting on the basal lamina. Astrocyte endfeet cover the majority of the vascular wall. Astrocyte processes surround synapses as well, and astrocytes play a central role in coupling the endothelial cells and neurons^46^. As such, astrocytes in the NVU can be considered the gatekeepers between the periphery and the nervous system. Astrocytes and endothelial cells participate in crosstalk that regulates permeability of the endothelial cell barrier by modulation of membrane transport proteins (GABA, norepinephrine and amino acid transporters), ion transporters, and tight junction proteins^47^. This crosstalk is mediated by multiple signaling molecules (including transforming growth factor-beta, basic fibroblast growth factor, glial-derived neurotrophic factor, and angiopoietin-1), their cognate receptors, and changes in gene expression^46^. While the functional phenotype of our splenic NCC is not clear, their anatomic location and constellation of surface markers strongly suggests an astrocyte-like identity and a putative role for these cells in controlling splenic vascular tone and/or permeability or lymphocyte trafficking.

The control of cell movement between compartments in the spleen is complex^48^. The splenic MZ is located at the border of the white pulp and the red pulp. The arterial circulation of the spleen terminates in the marginal sinus, within the MZ. Here, MZ B-lymphocytes readily encounter blood-borne pathogens and are able to rapidly initiate an immune response^36^. B-lymphocyte localization to the MZ requires expression of the Sphingosine-1-Phosphate Receptor 1 (S1P1)^37^. In the thymus, Sphingosine-1-Phosphate (S1P) ligand is produced by NCC-derived pericytes, and mediates lymphocyte egress^27^. Additionally, in the CNS, astrocytes produce S1P through activity of Sphingosine Kinase 1 (SphK1) and alterations in this pathway are involved in the pathogenesis of inflammatory CNS conditions, such as Multiple Sclerosis^49^. We have previously noted alterations in splenic B-lymphocyte populations in *EdnrB*^*NCC*−/−^ animals just prior to HAEC and here we observed decreased expression of S1P1 at the same time point. Together, our findings suggest that the process of B lymphocyte maturation and trafficking to the marginal zones may be altered in the *EdnrB*^*NCC*−/−^ spleen. Further work is needed to identify the precise cellular source of S1P in the spleen, and to delineate a potential role for splenic NCC in regulating lymphocyte trafficking in our HSCR/HAEC model.

A subpopulation of NCC migrate along nerve fibers of the peripheral nervous system and give rise to Schwann cells or neurons^50^. Once these cells commit to a Schwann cell lineage, they are termed Schwann cell precursors (SCP) and can give rise to Schwann cells, fibroblasts, melanocytes, parasympathetic neurons and enteric neurons^51^,^52^,^53^,^54^. These SCP rely on the extrinsic innervation for migration, and therefore appear to contribute to a later group of NCC to colonize the intestine^55^. Desert hedgehog (Dhh) is commonly employed to identify the SCP subset of NCC. Within the gut and mesentery, SCP express the glial markers BLBP and Sox10, and eventually express Tuj1 as they undergo neuronal differentiation, raising the possibility that some splenic NCC cells that we have observed are SCP-derived. While splenic NCC do not appear to be neurons (HuC/D-negative) and we have not observed any Sox10- or MAG-positive immunostained cells in post-natal spleens, recent studies have utilized conditional labeling with Desert hedgehog (Dhh)-Cre to identify SCP^53^. Therefore, the possibility remains that some splenic NCC derive from SCP.

Multiple pathophysiologic mechanisms for aganglionosis in HSCR have been advanced in the literature^14^. These include NCC-specific defects in proliferation, differentiation, migration and absolute cell numbers^56^. In the present study, we have demonstrated in the *EdnrB*^*NCC*−/−^ model that there does not appear to be alteration in the density of NCC entering the spleen prenatally, nor are there differences in splenic size in the early post-natal period. Together, these data suggest that the absolute numbers of NCC entering the spleen are conserved between genotypes. In order to understand the implications of this finding on the development of the ENS, which is primarily of vagal origin, further experiments to determine the precise axial origin of splenic NCC are needed.

In summary, we have demonstrated a novel contribution of NCC to the developing spleen. Splenic NCC display an astrocyte-like phenotype. We have also observed alterations in trafficking molecules, which appears to correlate with our previous lymphocyte observations in our murine model of HAEC. The identification of NCC-derived cells in the spleen provides a platform for further interrogation of the cellular and molecular mechanisms which underlie the neuro-immune response to inflammation.

## Methods

### Animals and Tissue Collection

Murine procedures were approved by the University of Wisconsin-Madison and University of Tennessee Health Science Center Animal Care and Use Committees. Avian procedures were approved at the Instituto de Biomedicina y Biotechnologia de Cantabria, Santander, Spain. *Macaca mulatta* procedures were approved by the Wisconsin National Primate Research Center. All experiments were performed under established guidelines for humane use and care of laboratory animals.

Mice carrying a NCC specific deletion of Endothelin Receptor B (*EdnrB*^*flex3/flex3*^) were mated with *TgWnt1-Cre*/+; *Rosa26*^*floxStop/tdTomato*^ animals to produce animals with either a heterozygous (*EdnrB*^*flex3*/+^) or homozygous deletion of *EdnrB (EdnrB*^*flex3/flex3*^). Throughout the manuscript these mice will be described as *EdnrB*^*NCC*+/−^ and *EdnrB*^*NCC*−/−^, respectively. In addition, all of the NCC within these animals were identified by their expression of the fluorescent protein tdTomato. *EdnrB*^*NCC*+/−^ mice are available from Jackson Laboratories (Stock Number: 009063). Male and Female *EdnrB*^*NCC*+/−^ animals were time mated and the day the vaginal plug was defined as embryonic day (E) 0.5. Embryos were isolated from pregnant dams that had been anesthetized with isoflurane and euthanized by cervical dislocation. Postnatal (P) animals were euthanized using isoflurane and cervical dislocation.

For weight determinations, murine spleens were isolated from P0, P9 and P18 animals (n = 3–6/genotype/time point). Murine gastrointestinal (GI) tracts and spleens were isolated from E13.5, E16.5 and E17.5 whole embryos and fixed in 4% paraformaldehyde (PFA) for 2 hours at room temperature (RT), while P0 and P21 GI tract and spleens were fixed in PFA for 4 hours at RT. All dissected tissues were then rinsed in 1x phosphate buffered saline (PBS) three times for 20 minutes on a rocking platform before being processed into 30% sucrose containing 0.1% sodium azide and stored at 4 °C until required^57^. Tissues were either immunostained as whole-mounts or processed into OCT and frozen for cryosectioning.

Transgenic chicken eggs with ubiquitous expression of GFP^58^ were obtained from the breeding colony established at the Instituto de Biomedicina y Biotechnologia de Cantabria in Santander, Spain. Eggs were incubated at 38 °C until day 19. Embryos were removed from the shell and the gastrointestinal tracts along with the spleen were isolated, fixed overnight at 4 °C in 4% PFA before being processed into 30% sucrose containing 0.1% sodium azide for cryosectioning.

Juvenile male Rhesus macaque (*Macaca mulatta*) spleens were obtained from the Nonhuman Primate Biological Materials Distribution Program of the Wisconsin National Primate Research Center at the University of Wisconsin-Madison and processed as described above for cryosectioning.

### Immunohistochemistry

Whole-mount tissues were rinsed in 1x PBS for 20 minutes three times on a rocking platform to wash out the 30% sucrose, then placed into blocking solution (1xPBS with 10% heat inactivated sheep serum with 0.1% Triton X-100) for 2 hours at RT on a rocking platform. They were then incubated overnight at 4°C with the appropriate primary antibodies diluted in blocking solution. Rat anti-CD31 (550274, BD Pharmingen, San Jose, CA; 1:100), Rat anti-PDGFRb (14-1402-81, eBioscience, San Deigo, CA; 1:100), mouse anti-TuJ1 (MMS 435 P, Covance, Madison, WI; 1:1000), rabbit anti-BLBP (ab32423, abcam, Cambridge, MA; 1:400), rabbit anti-S100 (Z0311, Dako, Carpinteria, CA; 1:300), rabbit anti-GFAP (Z033, Dako, Carpinteria, CA; 1:500), mouse anti-Myelin Associated Glyocprotein (MAG) (MAB1567, EMD Millipore, Darmstadt, Germany, 1:200), goat anti-Sox10 (SC-17342, Santa Cruz Biotechnology, Dallas, TX), mouse anti-PGP9.5 (MCA4750GA, Bio-Rad, Hercules, CA), and human anti-HuC/D (gift from Dr. Miles L. Epstein, Madison, WI). The following day, they were rinsed in 1x PBS (3 × 1 hour) on a rocking platform and finally a 1:500 dilution of secondary antibodies was added in blocking solution overnight at 4 °C. (Alexa Fluor 488 donkey anti-rat {A21208, Life Technologies, Grand Island, NY}, Alexa Fluor 488 goat anti-mouse {A11001, Life Technologies, Grand Island, NY}, Alexa Fluor donkey anti-rabbit 488 {711-545-152, Jackson ImmunoResearch, West Grove, PA}, Alexa Fluor donkey anti-mouse 488 {A-21202, ThermoFisher Scientific, Waltham, MA}, Alexa Fluor donkey anti-goat {A-11055, ThermoFisher Scientific, Waltham, MA}, Alexa Fluor donkey anti-human {709-545-149, Jackson ImmunoResearch, West Grove, PA}). The tissues were rinsed in 1xPBS for one hour three times and mounted onto glass slides in Fluoromount G with DAPI (Southern Biotech, Birmingham, AL).

16 μm cryosections of the spleens from mice, chick and juvenile monkeys were immunostained in a similar manner, except that the primary antibody incubation was performed at RT for 2 hours and the secondary antibody solution was added for 1 hour at RT. The slides were then mounted with Fluoromount G with DAPI.

### Image analysis

Low magnification bright field images of GI tracts and spleens were captured on a Nikon SMZ1500 stereoscope. Immunostained tissues and cryosections were imaged using a Nikon A1 confocal microscope. Nikon Elements software (Nikon, Melville, NY, USA) was used to capture Z-series. The brightness and contrast may have been adjusted for clarity with Photoshop^TM^ software (Adobe, USA). For determination of splenic NCC density, murine spleens were isolated from P0 and P21 (n = 3–6/genotype/time point).

### Semi-quantitative RT PCR

Spleens were dissected from P21 animals and total RNA was extracted using TRIzol Reagent (Life Technologies, Grand Island, NY). 2 μg of total RNA was used to transcribe cDNA using MultiScribe^TM^ Reverse Transcriptase (Life Technologies, Grand Island, NY). PCR was performed using Taq DNA polymerase (Life Technologies, Grand Island, NY) with primers specific for S1P1 and β-actin^59^. Samples were analyzed in triplicate from 3 different P21 mouse spleens. Quantification was performed by normalizing the expression level of *S1P1* in each sample to β-actin using densitometric analysis in ImageJ software (National Institutes of Health, USA).

### Statistical analysis

The data are shown as the mean values ± standard error of the mean (SEM). Unpaired Student’s T-tests were performed for statistical analysis using Microsoft Excel (Microsoft, USA) and *p*-values < 0.05 were considered significant.

## Additional Information

**How to cite this article**: Barlow-Anacker, A. J. *et al*. Neural Crest Cells Contribute an Astrocyte-like Glial Population to the Spleen. *Sci. Rep.* **7**, 45645; doi: 10.1038/srep45645 (2017).

**Publisher's note:** Springer Nature remains neutral with regard to jurisdictional claims in published maps and institutional affiliations.

## Supplementary Material

Supplementary Figures

Supplementary Movie

## References

[b1] Ji H. . Central cholinergic activation of a vagus nerve-to-spleen circuit alleviates experimental colitis. Mucosal Immunology 7, 335–347 (2013). 23881354 10.1038/mi.2013.52 PMC3859808

[b2] van der Zanden E. P. . Vagus nerve activity augments intestinal macrophage phagocytosis via nicotinic acetylcholine receptor alpha4beta2. Gastroenterology 137, 1029–39– 1039.e1–4 (2009). 19427310 10.1053/j.gastro.2009.04.057

[b3] Mina-Osorio P. . Neural signaling in the spleen controls B-cell responses to blood-borne antigen. Mol. Med. 18, 618–627 (2012). 22354214 10.2119/molmed.2012.00027 PMC3388134

[b4] Bronner M. E. & LeDouarin N. M. Development and evolution of the neural crest: An overview. Developmental Biology 366, 2–9 (2012). 22230617 10.1016/j.ydbio.2011.12.042 PMC3351559

[b5] Takahashi Y., Sipp D. & Enomoto H. Tissue Interactions in Neural Crest Cell Development and Disease. Science 341, 860–863 (2013). 23970693 10.1126/science.1230717

[b6] Rühl A. Glial cells in the gut. Neurogastroenterol. Motil. 17, 777–790 (2005). 16336493 10.1111/j.1365-2982.2005.00687.x

[b7] Bush T. G. Enteric glial cells. An upstream target for induction of necrotizing enterocolitis and Crohn’s disease? Bioessays 24, 130–140 (2002). 11835277 10.1002/bies.10039

[b8] Spadoni I. . A gut-vascular barrier controls the systemic dissemination of bacteria. Science 350, 830–834 (2015). 26564856 10.1126/science.aad0135

[b9] Isern J. . The neural crest is a source of mesenchymal stem cells with specialized hematopoietic stem cell niche function. eLife 3, e03696 (2014). 25255216 10.7554/eLife.03696 PMC4381911

[b10] Yamazaki H. Presence and distribution of neural crest-derived cells in the murine developing thymus and their potential for differentiation. International Immunology 17, 549–558 (2005). 15837714 10.1093/intimm/dxh237

[b11] Foster K. . Contribution of neural crest-derived cells in the embryonic and adult thymus. J. Immunol. 180, 3183–3189 (2008). 18292542 10.4049/jimmunol.180.5.3183

[b12] Komada Y. . Origins and properties of dental, thymic, and bone marrow mesenchymal cells and their stem cells. PLoS ONE 7, e46436 (2012). 23185234 10.1371/journal.pone.0046436 PMC3504117

[b13] Zachariah M. A. & Cyster J. G. Neural crest-derived pericytes promote egress of mature thymocytes at the corticomedullary junction. Science 328, 1129–1135 (2010). 20413455 10.1126/science.1188222 PMC3107339

[b14] Bondurand N. & Southard-Smith E. M. Mouse models of Hirschsprung disease and other developmental disorders of the enteric nervous system: Old and new players. Developmental Biology 417, 139–157 (2016). 27370713 10.1016/j.ydbio.2016.06.042 PMC5026931

[b15] Amiel J. . Hirschsprung disease, associated syndromes and genetics: a review. J. Med. Genet. 45, 1–14 (2008). 17965226 10.1136/jmg.2007.053959

[b16] Gosain A. Established and emerging concepts in Hirschsprung’s-associated enterocolitis. Pediatr. Surg. Int. 32, 313–320 (2016). 26783087 10.1007/s00383-016-3862-9 PMC5321668

[b17] Cheng Z. . Splenic lymphopenia in the endothelin receptor B-null mouse: implications for Hirschsprung associated enterocolitis. Pediatr. Surg. Int. 27, 145–150 (2010). 10.1007/s00383-010-2787-y PMC3755962 21046116

[b18] Gosain A. . Impaired Cellular Immunity in the Murine Neural Crest Conditional Deletion of Endothelin Receptor-B Model of Hirschsprung’s Disease. PLoS ONE 10, e0128822 (2015). 26061883 10.1371/journal.pone.0128822 PMC4465674

[b19] Druckenbrod N. R., Powers P. A., Bartley C. R., Walker J. W. & Epstein M. L. Targeting of endothelin receptor-B to the neural crest. genesis 46, 396–400 (2008). 18693272 10.1002/dvg.20415 PMC2610478

[b20] Pierre J. F. . Intestinal dysbiosis and bacterial enteroinvasion in a murine model of Hirschsprung’s disease. J. Pediatr. Surg. 49, 1242–1251 (2014). 25092084 10.1016/j.jpedsurg.2014.01.060 PMC4122863

[b21] Zaitoun I. . Altered neuronal density and neurotransmitter expression in the ganglionated region of Ednrbnull mice: implications for Hirschsprung’s disease. Neurogastroenterology & Motility 25, e233–e244 (2013). 23360229 10.1111/nmo.12083 PMC3578114

[b22] Erickson C. S. . Sacral neural crest-derived cells enter the aganglionic colon of Ednrb−/− mice along extrinsic nerve fibers. J. Comp. Neurol. 520, 620–632 (2011). 10.1002/cne.22755 PMC3500027 21858821

[b23] Frykman P. K., Cheng Z., Wang X. & Dhall D. Enterocolitis causes profound lymphoid depletion in endothelin receptor B- and endothelin 3-null mouse models of Hirschsprung-associated enterocolitis. Eur. J. Immunol. 45, 807–817 (2015). 25487064 10.1002/eji.201444737 PMC4370321

[b24] Dang R. . Lymphopenia in Ednrb-deficient rat was strongly modified by genetic background. Biomed. Res. 33, 249–253 (2012). 22975636 10.2220/biomedres.33.249

[b25] Druckenbrod N. R. & Epstein M. L. Age-dependent changes in the gut environment restrict the invasion of the hindgut by enteric neural progenitors. Development 136, 3195–3203 (2009). 19700623 10.1242/dev.031302

[b26] Barlow A. J., Wallace A. S., Thapar N. & Burns A. J. Critical numbers of neural crest cells are required in the pathways from the neural tube to the foregut to ensure complete enteric nervous system formation. Development 135, 1681–1691 (2008). 18385256 10.1242/dev.017418

[b27] Cyster J. G. & Schwab S. R. Sphingosine-1-Phosphate and Lymphocyte Egress from Lymphoid Organs. Annu. Rev. Immunol. 30, 69–94 (2012). 22149932 10.1146/annurev-immunol-020711-075011

[b28] Erickson C. S. . Appearance of cholinergic myenteric neurons during enteric nervous system development: comparison of different ChAT fluorescent mouse reporter lines. Neurogastroenterology & Motility 26, 874–884 (2014). 24712519 10.1111/nmo.12343 PMC4037379

[b29] Jessen K. R. & Mirsky R. The origin and development of glial cells in peripheral nerves. Nat Rev Neurosci 6, 671–682 (2005). 16136171 10.1038/nrn1746

[b30] Finzsch M. . Sox10is required for Schwann cell identity and progression beyond the immature Schwann cell stage. The Journal of Cell Biology 189, 701–712 (2010). 20457761 10.1083/jcb.200912142 PMC2872908

[b31] Kuhlbrodt K., Herbarth B., Sock E., Hermans-Borgmeyer I. & Wegner M. Sox10, a novel transcriptional modulator in glial cells. J. Neurosci. 18, 237–250 (1998). 9412504 10.1523/JNEUROSCI.18-01-00237.1998 PMC6793382

[b32] Delalande J. M., Thapar N. & Burns A. J. Dual labeling of neural crest cells and blood vessels within chicken embryos using Chick(GFP) neural tube grafting and carbocyanine dye DiI injection. J Vis Exp e52514, doi: 10.3791/52514 (2015). 26065540 PMC4542995

[b33] Tucker G. C., Aoyama H., Lipinski M., Tursz T. & Thiery J. P. Identical reactivity of monoclonal antibodies HNK-1 and NC-1: conservation in vertebrates on cells derived from the neural primordium and on some leukocytes. Cell Differ. 14, 223–230 (1984). 6207939 10.1016/0045-6039(84)90049-6

[b34] Sofroniew M. V. & Vinters H. V. Astrocytes: biology and pathology. Acta Neuropathol. 119, 7–35 (2010). 20012068 10.1007/s00401-009-0619-8 PMC2799634

[b35] Balaskas C. & Gabella G. Glial fibrillary acidic protein (GFAP) immunoreactivity in enteric ganglia of the chick embryo. Brain Res. 804, 275–283 (1998). 9757063 10.1016/s0006-8993(98)00709-4

[b36] Cerutti A., Cols M. & Puga I. Marginal zone B cells: virtues of innate- like antibody-producing lymphocytes. Nature Publishing Group 13, 118–132 (2013). 10.1038/nri3383 PMC3652659 23348416

[b37] Cinamon G. . Sphingosine 1-phosphate receptor 1 promotes B cell localization in the splenic marginal zone. Nature Immunology 5, 713–720 (2004). 15184895 10.1038/ni1083

[b38] Segura B. J. . Sphingosine-1-phosphate mediates calcium signaling in guinea pig enteroglial cells. Journal of Surgical Research 116, 42–54 (2004). 14732348 10.1016/s0022-4804(03)00281-6

[b39] Müller S. M. . Neural crest origin of perivascular mesenchyme in the adult thymus. J. Immunol. 180, 5344–5351 (2008). 18390716 10.4049/jimmunol.180.8.5344

[b40] Plank J. L. . Influence and timing of arrival of murine neural crest on pancreatic beta cell development and maturation. Developmental Biology 349, 321–330 (2011). 21081123 10.1016/j.ydbio.2010.11.013 PMC3019241

[b41] Nagoshi N. . Ontogeny and Multipotency of Neural Crest-Derived Stem Cells in Mouse Bone Marrow, Dorsal Root Ganglia, and Whisker Pad. Cell Stem Cell 2, 392–403 (2008). 18397758 10.1016/j.stem.2008.03.005

[b42] Morikawa S. . Development of mesenchymal stem cells partially originate from the neural crest. Biochemical and Biophysical Research Communications 379, 1114–1119 (2009). 19161980 10.1016/j.bbrc.2009.01.031

[b43] Neuwelt E. A. . PeRSPeCTiveS. 1–14, doi: 10.1038/nrn2995 (2011).

[b44] Stanimirovic D. B. & Friedman A. Pathophysiology of the neurovascular unit: disease cause or consequence? 32, 1207–1221 (2012). 10.1038/jcbfm.2012.25 PMC3390807 22395208

[b45] Gordon G. R. J., Mulligan S. J. & MacVicar B. A. Astrocyte control of the cerebrovasculature. Glia 55, 1214–1221 (2007). 17659528 10.1002/glia.20543

[b46] Abbott N. J., Rönnbäck L. & Hansson E. Astrocyte–endothelial interactions at the blood–brain barrier. Nat Rev Neurosci 7, 41–53 (2006). 16371949 10.1038/nrn1824

[b47] Lee S.-W. . SSeCKS regulates angiogenesis and tight junction formation in blood-brain barrier. Nature Medicine 9, 900–906 (2003). 10.1038/nm889 12808449

[b48] Arnon T. I. . GRK2-Dependent S1PR1 Desensitization Is Required for Lymphocytes to Overcome Their Attraction to Blood. Science 333, 1898–1903 (2011). 21960637 10.1126/science.1208248 PMC3267326

[b49] Krone B. & Grange J. M. Paradigms in multiple sclerosis: time for a change, time for a unifying concept. Inflammopharmacology 19, 187–195 (2011). 21547536 10.1007/s10787-011-0084-6 PMC3127006

[b50] Coppola E. . Epibranchial ganglia orchestrate the development of the cranial neurogenic crest. Proceedings of the National Academy of Sciences 107, 2066–2071 (2010). 10.1073/pnas.0910213107 PMC2836672 20133851

[b51] Dyachuk V. . Neurodevelopment. Parasympathetic neurons originate from nerve-associated peripheral glial progenitors. Science 345, 82–87 (2014). 24925909 10.1126/science.1253281

[b52] Espinosa-Medina I. . Neurodevelopment. Parasympathetic ganglia derive from Schwann cell precursors. Science 345, 87–90 (2014). 24925912 10.1126/science.1253286

[b53] Uesaka T., Nagashimada M. & Enomoto H. Neuronal Differentiation in Schwann Cell Lineage Underlies Postnatal Neurogenesis in the Enteric Nervous System. Journal of Neuroscience 35, 9879–9888 (2015). 26156989 10.1523/JNEUROSCI.1239-15.2015 PMC6605410

[b54] Adameyko I. . Schwann cell precursors from nerve innervation are a cellular origin of melanocytes in skin. Cell 139, 366–379 (2009). 19837037 10.1016/j.cell.2009.07.049

[b55] Uesaka T., Young H. M., Pachnis V. & Enomoto H. Development of the intrinsic and extrinsic innervation of the gut. Developmental Biology 417, 158–167 (2016). 27112528 10.1016/j.ydbio.2016.04.016

[b56] Lake J. I. & Heuckeroth R. O. Enteric nervous system development: migration, differentiation, and disease. AJP: Gastrointestinal and Liver Physiology 305, G1–G24 (2013). 10.1152/ajpgi.00452.2012 PMC3725693 23639815

[b57] Barlow-Anacker A. J., Erickson C. S., Epstein M. L. & Gosain A. Immunostaining to visualize murine enteric nervous system development. J Vis Exp e52716, doi: 10.3791/52716 (2015). 25993536 PMC4541606

[b58] McGrew M. J. . Efficient production of germline transgenic chickens using lentiviral vectors. EMBO Rep. 5, 728–733 (2004). 15192698 10.1038/sj.embor.7400171 PMC1299092

[b59] Matloubian M. . Lymphocyte egress from thymus and peripheral lymphoid organs is dependent on S1P receptor 1. Nature 427, 355–360 (2004). 14737169 10.1038/nature02284

