# Supplementary material for: Neural Crest Cells Contribute an Astrocyte-like Glial Population to the Spleen
Source: Sci Rep. 2017 Mar 28;7:45645. doi: 10.1038/srep45645 (PMC5368681; doi:10.1038/srep45645)
Supplement: Supplementary Figures [file srep45645-s1.pdf]

Title: Neural Crest Cells Contribute an Astrocyte-like Glial Population to the Spleen

Authors: Amanda J. Barlow-Anacker, PhD[1], Ming Fu, MD, PhD[2], Christopher S. Erickson, BS[1], Federica Bertocchini, PhD[3], Ankush Gosain, MD, PhD[2,4,*]

Affiliations:

[1] Department of Surgery, University of Wisconsin School of Medicine and Public Health, Madison, Wisconsin, United States of America.

[2] Division of Pediatric Surgery, Department of Surgery, University of Tennessee Health Sciences Center, Memphis, Tennessee, United States of America.

[3] Instituto de Biomedicina y Biotechnologia de Cantabria, Santander, Spain[4] Children's Foundation Research Institute, Le Bonheur Children's Hospital, Memphis, Tennessee, United States of America.

Figure S1

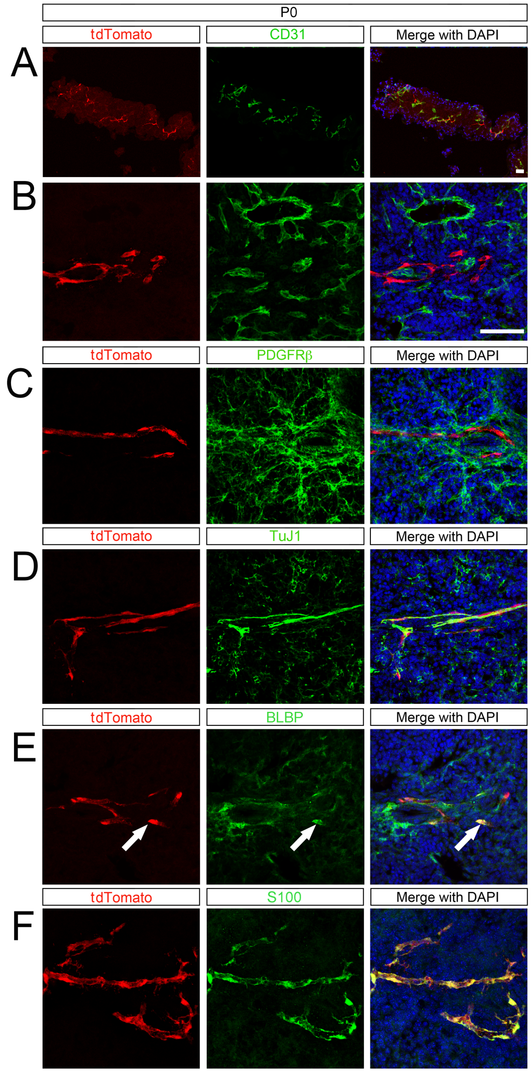

Supplementary Figure 1. NCC within the spleen at P0 express glial markers. (A) tdTomato NCC are seen in an arborized pattern near the vasculature (CD31) of the spleen. (B) tdTomato NCC are found outside of blood vessels (CD31) and (C) their surrounding pericytes (PDGFRβ). (D) tdTomato NCC are associated with nerve fibers (TuJ1). (E) By P0, all tdTomato NCC express BLBP (early glial lineage) and (F) S100-β (glial lineage). Arrowheads (white) highlight double-labeled cells. Scale bar: 20μm.

Figure S2

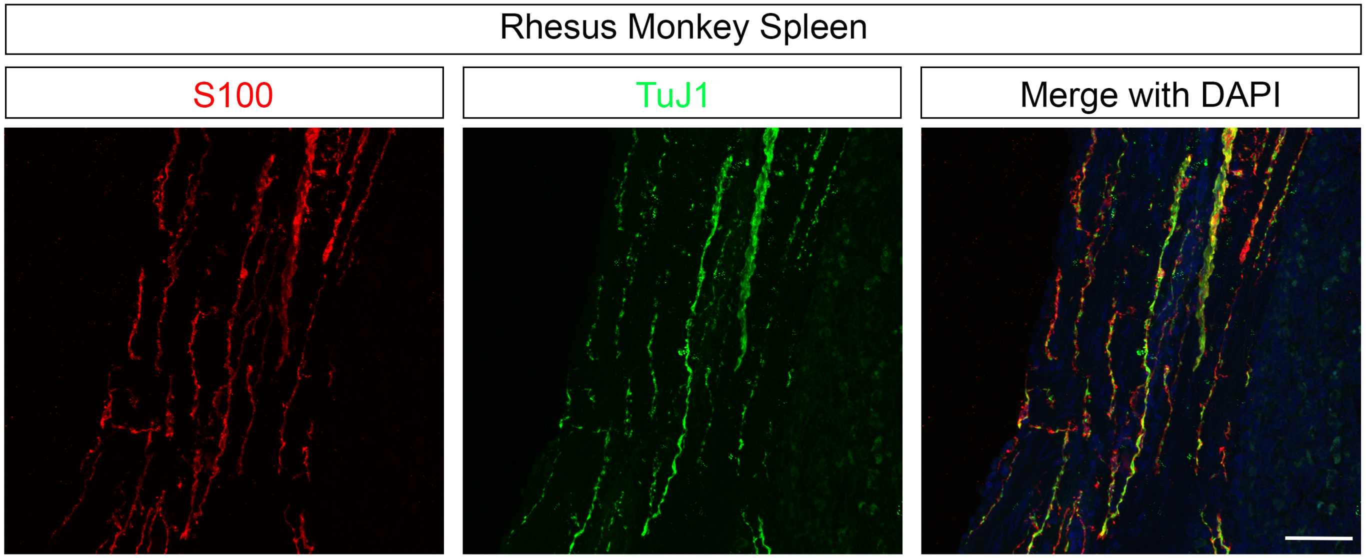

Supplementary Figure 2. Splenic NCC in non-human primates are associated with nerve fibers. Within the adolescent rhesus monkey spleen, glial cells (S100-β, red) are found in proximity to TuJ1+ nerve fibers (green). See also Supplementary movie. Scale bar: 50μm.

Figure S3

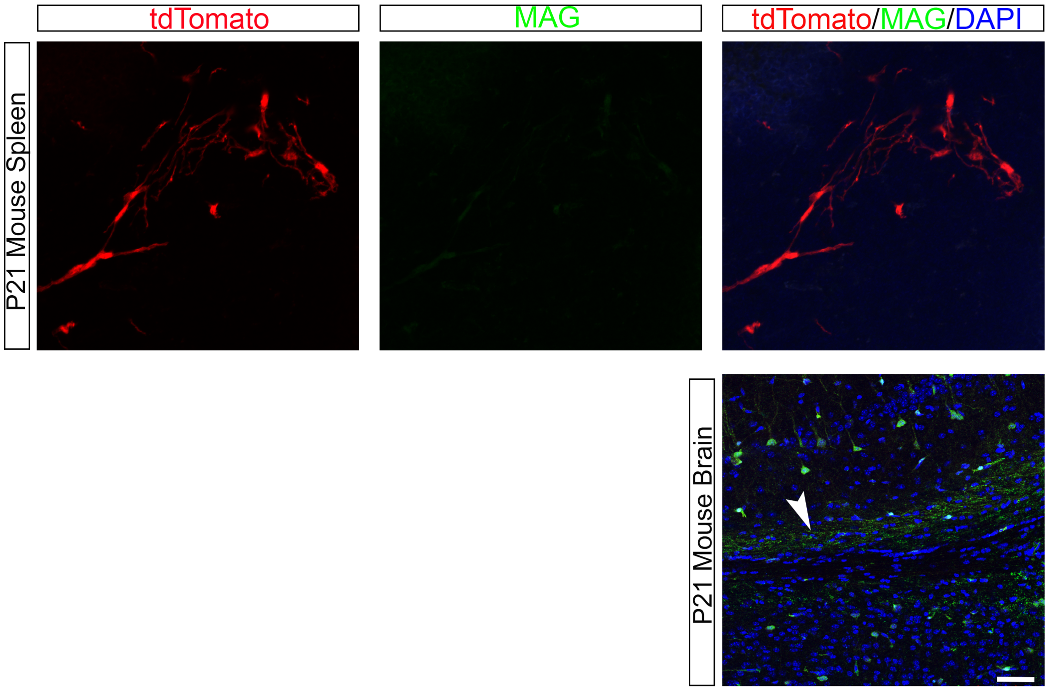

Supplementary Figure 3. Splenic NCC do not express myelin-associated glycoprotein (MAG). Top: In the P21 murine spleen, NCC (tdTomato) do not demonstrate staining for MAG (green), which can be used to identify myelinated Schwann Cells. Bottom: MAG+ cells (green) are seen in the white matter of the murine brain (arrowhead). Scale bar: 50μm.

Figure S4

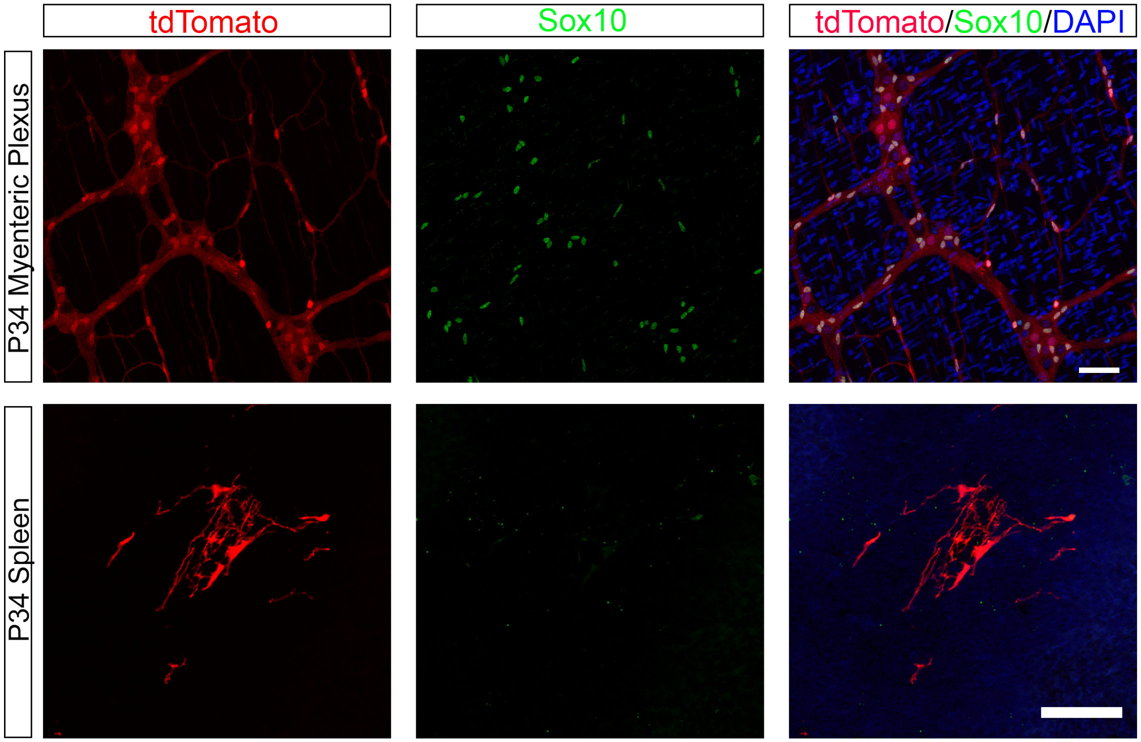

Supplementary Figure 4. Splenic NCC do not express Sox10. Top: NCC-dervied (tdTomato) glial cells within the myenteric plexus of the intestine are Sox10+ (green). Bottom: NCC (tdTomato) in the spleen do not demonstrate Sox10. Scale bar: 50μm.

Figure S5

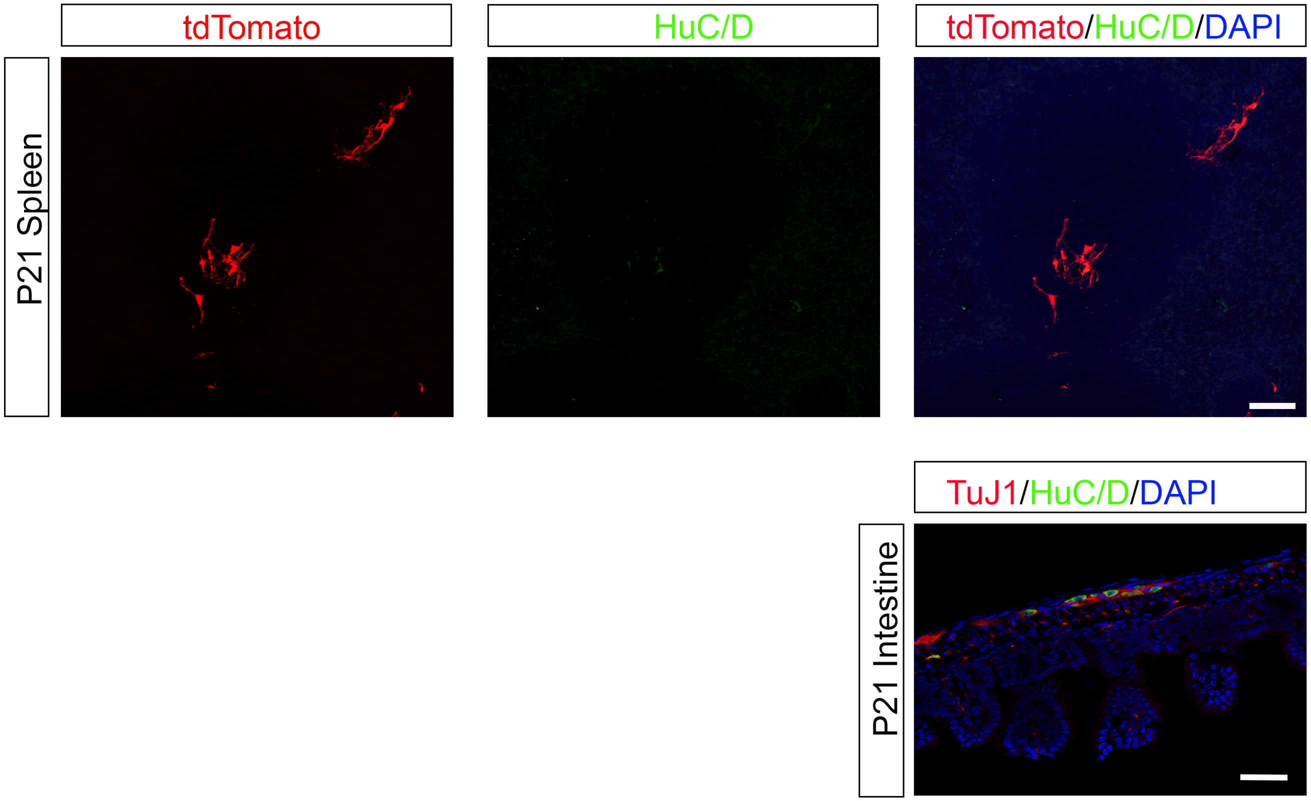

Supplementary Figure 5. Splenic NCC do not express HuC/D. Top: NCC (tdTomato) in the spleen do not demonstrate HuC/D immunoreactivity. Bottom: HuC/D (green) identifies neuronal cell bodies, seen with TuJ1+ (red) nerve fibers in the myenteric plexus of the post-natal intestine. Scale bar: 50μm.

Supplementary Movie

Supplementary Movie. Splenic NCC in the adolescent rhesus monkey are associated with nerve fibers. 3D reconstruction of confocal imaging. Within the adolescent rhesus monkey spleen, glial cells (S100-β, red) are found in proximity to TuJ1+ nerve fibers (green).
